# Planarian Mucus: A Novel Source of Pleiotropic Cytotoxic and Cytostatic Agents against Cancer Cells

**DOI:** 10.3390/biom14091075

**Published:** 2024-08-28

**Authors:** Gaetana Gambino, Eleonora Da Pozzo, Alessandra Salvetti, Leonardo Rossi

**Affiliations:** 1 Department of Clinical and Experimental Medicine, Via Volta 4, 56126 Pisa, Italy; gaetana.gambino@unipi.it (G.G.); leonardo.rossi@unipi.it (L.R.); 2 Department of Pharmacy, Via Bonanno 6, 56126 Pisa, Italy; eleonora.dapozzo@unipi.it

**Keywords:** planarian, mucus, anticancer activity, lipid droplets, mitochondria, reactive oxygen species

## Abstract

Biological evolution has generated a vast array of natural compounds produced by organisms across all domains. Among these, secondary metabolites, selected to enhance an organism’s competitiveness in its natural environment, make them a reservoir for discovering new compounds with cytotoxic activity, potentially useful as novel anticancer agents. Slime secretions, the first barrier between epithelial surfaces and the surrounding environment, frequently contain cytotoxic molecules to limit the growth of parasitic organisms. Planarians, freshwater Triclads, continuously secrete a viscous mucus with multiple physiological functions. The chemical composition of planarian mucus has been only partially elucidated, and there are no studies reporting its cytotoxic or cytostatic effects. In this study, we developed a protocol for collecting mucus from *Dugesia japonica* specimens and we demonstrated that it inhibits the growth of cancer cells by activating cytostatic and ROS-dependent cytotoxic mechanisms inducing lipid droplet accumulation and mitochondrial membrane reorganization. Although further research is needed to identify the specific chemicals responsible for the anticancer activity of planarian mucus, this work opens up numerous research avenues aimed at better understanding the mechanisms of action of this product for potential therapeutic applications.

## 1. Introduction

Natural products, organic compounds synthesized by living organisms, historically played a pivotal role in the development of new drugs, with more than 60% of current anticancer drugs relying on molecules of natural origin [1]. The reason is that the complexity of biological evolution is manifest not only in the myriad of living species present in nature but also in the immense variety of organic natural products generated by them [2]. Moreover, natural products exhibit high structural diversity, far exceeding the capabilities of synthetic organic chemists, and are the result of an evolutionary process that spans hundreds of thousands of years, shaping their utility for unique biologically relevant functions. In contrast to synthesized chemical compounds, natural products exhibit higher molar weight, a notable presence of carbon and oxygen atoms and fewer nitrogen atoms and halogens, a higher number of H-bond acceptors and donors, higher hydrophilicity and greater molecular rigidity [3,4]. Most bioactive natural products are secondary metabolites, which are not essential for survival and growth but increase the competitiveness of the organism within its environment. They may function as defense mechanisms against other species, thus explaining the frequent anticancer and anti-infectious disease activities of natural compounds, relevant in both traditional and modern medicine [5].

The crucial role of natural products in the discovery and development of anticancer agents is widely recognized [6]. Despite several new natural products currently being investigated as potential cytotoxic agents, showing a positive trend in preclinical research [7], most cancers remain untreatable due to their high heterogeneity and the development of chemoresistance mechanisms. Thus, novel potential sources are constantly being explored for the discovery of innovative bioactive metabolites with anti-neoplastic properties.

The plant kingdom and marine organisms represent the most investigated sources due to the enormous number of species and their evolutionary adaptation to diverse environments [8,9,10], while limited research has been conducted on freshwater organisms.

Among these, planarians—flatworms belonging to the Platyhelminthes phylum, class Turbellaria, order Tricladida—are widely used as model systems in tissue regeneration, stem cell research, and developmental biology. This is due to their unique biological features, including remarkable regenerative abilities, a high abundance of stem cells, continuous cell turnover, and an absence of aging phenomena [11,12]. Large isogenic colonies of planarians can be easily maintained under standardized laboratory conditions at minimal cost, making them a reliable alternative to mammalian models for in vivo toxicological and pharmacological studies [13,14,15], without ethical concerns and with an almost limitless supply of specimens for large-scale experimental drug screening. These characteristics, combined with the discovery that a crude extract from a Maltese planarian species exhibits anticancer effects on the HL-60 AML human cell line in vitro [16], have sparked our interest in investigating the potential anticancer effects of planarian mucus on cancer cell growth. Indeed, freshwater planarians secrete a slime [17], a viscid mucus with multiple functions, including locomotion by ciliary gliding, lubricating the body surface, adhesion to the substrate, and prey capture. Moreover, as the first barrier between the single-layer planarian epidermis and the surrounding environment, its involvement in receiving tactile stimuli, protection, in innate immunity mechanisms and predator avoidance has been proposed [18,19,20]. The mucus trail left behind by the animal during locomotion represents a significant resource for bacterial growth and the establishment of a food web, serving as a valuable reservoir of bacterivorous prey for planarians themselves [21]. Mucus derives from specialized secretory granules known as rhabdites that, once secreted, swell on contact with water to form the viscous mucus.

Histochemical studies have revealed that the ejected slime contains protein, little lipid and is rich in sulphated glycosaminoglycans (sGAGs) [17,18,22,23]. The proteome of planarian slime shares significant similarities with human nasal mucus, olfactory mucus, cervical mucus, and tear fluid [24], and includes immune-associated proteins and antioxidant enzymes [19,24]. Despite being poorly investigated, the potential role of mucus in innate defense mechanisms provides a good premise for assuming its cytotoxic activity in accordance with other mucus secretions, such those of the terrestrial snails *Achatina fulica* and *Actinia equine* [25,26]. Thus, we developed a mucus extraction procedure and analyzed its effects on different tumor cell lines, focusing then on the human bronchioalveolar carcinoma cell line NCI-H358.

## 2. Materials and Methods

### 2.1. Planarian Rearing

Planarians of the species *Dugesia japonica* (clonal strain GI) were raised in planarian water following the method of Balestrini and colleagues [27], and were fed with chicken liver, provided once per week. Animals were starved for one week before being collected for mucus extraction. In some experiments, animals were treated with 100 units/mL penicillin and 100 μg/mL streptomycin for one week before mucus collection.

### 2.2. Planarian Mucus Preparation, Quantification, Storage and Treatment

**Figure 1:**
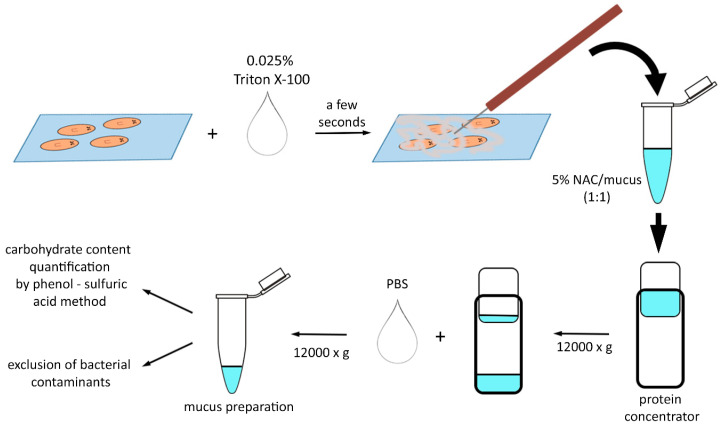
Scheme Depicting the Procedure Followed to Obtain Planarian Mucus.

*D. japonica* mucus was extracted and concentrated according to the scheme depicted in Figure 1. Approximately 200 specimens of planarians, each with a length between 5 and 6 mm, were placed on glass coverslips. The rearing water was removed, and the planarians were covered with a solution of 0.025% Triton X-100 diluted in planarian water. After a few seconds, the excreted mucus was manually collected using a sleeved needle, placed in a vial, measured, and dissolved in an equal volume of 5% N-acetylcysteine (NAC; Sigma-Aldrich). Pierce protein concentrators with a PES 3K MWCO (Thermo Fisher Scientific) were used to return the mucus to its original volume. The mucus solution was then diluted 10 times with PBS to achieve an NAC concentration of 0.25% and again concentrated to its original volume. The presence of bacterial contaminants capable of growing at 37 °C in the presence of 100 units/mL penicillin and 100 μg/mL streptomycin (Corning) (the same concentration used in cell culture media) was excluded by plating mucus aliquots on brain heart infusion agar (Millipore) plates. To verify the quality of the mucus preparation and standardize subsequent treatments, the concentration of polysaccharides was quantified using the phenol sulfuric acid method for carbohydrate estimation, as described by Masuko et al. [28]. After quantification, the mucus was diluted to 50 μg/mL in NAC 0.25% (15 mM NAC final concentration) and immediately used or stored in small aliquots at −80 °C. The planarians used to extract the mucus were not damaged by the procedure and could be reintegrated into the colony for future use. Cells treated with an equal volume of vehicle served as controls (i.e., for experiments with 0.4 μg/mL mucus final concentration, 8 μL/mL of mucus was used for treated samples and 8 μL/mL of 0.25% NAC was used in control samples to obtain a final concentration of 0.12 mM NAC).

### 2.3. Cell Cultures

The normal human bronchial epithelial cell line 16HBE14 was purchased from Sigma-Aldrich (code SCC150). The human non-small-cell lung cancer cell line A549 and the human bronchioalveolar carcinoma cell line NCI-H358 were kindly provided by Doctor Laura Poliseno from the Italian National Research Council of Pisa. The Caco-2 human colorectal adenocarcinoma epithelial cell line was provided by Professor Antonella Cecchettini from the Department of Clinical and Experimental Medicine at the University of Pisa. The THP-1 human leukemia monocytic cell line was provided by Professor Alessandro Corti from the Department of Translational Research and New Technologies in Medicine and Surgery.

NCI-H358, Caco-2, and THP-1 cells were cultured in RPMI 1640 medium w/o L-glutamine (yourSial), while A549 cells were cultured in DMEM (Sigma-Aldrich by Merck, Darmstadt, Germany) and 16HBE14o cells were cultured in EMEM (Corning, Corning, NY, USA). In all cases, the media were supplemented with 10% heat-inactivated fetal bovine serum (Corning), 2 mM L-glutamine, 100 units/mL penicillin, and 100 μg/mL streptomycin (Corning). Cells were cultured at 37 °C in a humidified atmosphere containing 5% CO_2_ and 95% air and subjected to a 1:3 split every 3 days.

### 2.4. Crystal Violet Assay

A total of 9000 cells were seeded in 96-well plates. After 24 h, the growth medium was replaced with fresh medium containing the desired treatment, and the cells were left to grow for an additional 24 or 48 h. Subsequently, the cells were washed twice with PBSCa (1 × PBS plus 1 mM CaCl_2_) and fixed in 10% buffered formalin for 10 min at room temperature. After two washes with PBSCa, the cells were incubated in crystal violet staining solution (0.5% crystal violet, C3886 Sigma-Aldrich/20% methanol) for 10 min. The crystal violet was then carefully removed, and the multi-well plates were washed three times by immersion in tap water. After complete drying, 0.1 mL of crystal violet destaining solution (50% ethanol/0.1 M sodium citrate, pH 4.2) was added to each well, and the optical density was measured by reading the absorbance at 540 nm. For each experimental group, three wells were measured, and absorbance values were corrected by subtracting the optical density of wells containing only the growth medium with added mucus, used as the blank.

### 2.5. Propidium Iodide Viability Assay

A total of 40,000 cells were seeded in 24-well plates. After 24 h, the growth medium was replaced with fresh medium containing the desired treatment, and the cells were left to grow for an additional 48 h. Both floating and adherent cells were harvested and stained with 2 µg/mL propidium iodide (PI) (P1304MP, Invitrogen by Thermo Fisher Scientific, Waltham, MA, USA) for 5 min. Stained cells were analyzed using an ACCURI C6 PLUS (BD Biosciences, Franklin Lakes, NJ, USA) flow cytometer. Doublet discrimination was manually conducted by plotting events on a linear scale based on the FSC signal area versus the FSC signal height. Gated events were then visualized on a linear scale based on the FSC signal area versus the FL3 670 LP channel signal area on a logarithmic scale to create a plot distinguishing dead (stained) cells from live (unstained) cells. Three independent samples were analyzed for each experimental class, and a minimum of 5000 events were acquired for each sample.

### 2.6. Cell Cycle Analysis

A total of 40,000 cells were seeded in 24-well plates. After 24 h, the growth medium was replaced with fresh medium containing the desired treatment, and the cells were left to grow for an additional 24 or 48 h. Analysis of the cell cycle was performed by staining cell DNA with 1 mg/mL propidium iodide (PI) (P1304MP, Invitrogen by Thermo Fisher Scientific) following RNA digestion.

Specifically, adherent cells were detached by trypsin treatment and counted using a hemocytometer. An equal number of cells for each sample was centrifuged, washed in PBS, and fixed with 3 volumes of cold ethanol. The cells were harvested by centrifugation, suspended in PI staining solution (PI 200 µg/mL Sigma-Aldrich/sodium citrate 0.1%/RNAse A 0.5 mg/mL/Nonidet NP40 0.1%) at a concentration of 200,000 cells/mL, and incubated overnight at 4 °C before cytofluorimetry analysis.

Stained cells were analyzed using an ACCURI C6 PLUS (BD Biosciences) flow cytometer. Doublet discrimination was manually conducted by plotting events on a linear scale based on the FSC signal area versus the FSC signal height. Gated events were plotted in the FL3 670 LP channel (linear) versus the FSC signal area or in the FL3 670 LP channel (linear) to produce a graph showing the distribution of cells in the cell cycle. Approximately 3000 events were acquired for each sample.

### 2.7. JC1 Assay

JC1 is a cationic carbocyanine probe that shows a potential-dependent accumulation in mitochondria. At low concentrations, it is in the form of monomers and emits green fluorescence (absorption/emission of 510/527 nm). At high concentrations, it aggregates and emits red fluorescence (absorption/emission 585/590 nm) [29]. Therefore, the red/green fluorescence intensity ratio is indicative of the mitochondrial transmembrane potential (ΔΨ).

A total of 40,000 cells were seeded in 24-well plates. After 24 h, the growth medium was replaced with fresh medium containing the desired treatment, and the cells were left to grow for an additional 24 or 48 h. Analysis of the mitochondrial transmembrane potential was performed by staining cells with the JC1 cationic dye (Thermo Fisher Scientific).

Specifically, adherent cells were detached by trypsin treatment and counted using a hemocytometer. A total of 1 × 10^5^ cells for each sample were centrifuged, suspended in 300 µL of PBS containing 20 µM JC1, incubated at 37 °C for 20 min, and immediately analyzed using the ACCURI C6 PLUS (BD Biosciences) cytofluorimeter. A positive control for complete depolarization was obtained by treating JC1-stained cells for 5 min with 50 µM CCCP (Sigma-Aldrich).

Doublet discrimination was obtained by plotting events on a linear scale according to the FSC signal area versus the FSC signal height. Gated events were then visualized on a logarithmic scale in both FL2 585 BP and FL1 530 BP channels to produce a plot for discriminating polarized and dissipated mitochondria. Approximately 3000 events were acquired for each sample.

### 2.8. Annexin V/PI Assay

A total of 40,000 cells were seeded in 24-well plates. After 24 h, the growth medium was replaced with fresh medium containing the desired treatment, and the cells were left to grow for an additional 48 h. Analysis of the externalization of phosphatidylserine was performed by staining cells with the FITC Annexin V Apoptosis Detection Kit I (BD Bioscience), following the manufacturer’s instructions. Stained cells were analyzed using the ACCURI C6 PLUS (BD Biosciences) cytofluorimeter. Following doublet discrimination, gated events were then visualized on a logarithmic scale in both FL3 670 LP and FL1 530 BP channels to produce a plot for discriminating early apoptotic cells (annexin V positive/PI negative), late apoptotic/secondary necrotic cells (annexin V positive/PI positive), and necrotic cells (annexin V negative/PI positive). Approximately 3000 events were acquired for each sample.

### 2.9. Analysis of Lipid Content

A total of 40,000 cells were seeded in 24-well plates. After 24 h, the growth medium was replaced with fresh medium containing the desired treatment, and the cells were left to grow for an additional 24 or 48 h. Analysis of the lipid content was performed by staining cells with Nile red. Stock solutions were prepared by dissolving Nile red powder (Sigma-Aldrich) in DMSO to a final concentration of 2 mM. Adherent cells were detached by trypsin treatment, suspended in 300 µL of PBS containing 0.5 µM Nile red, and incubated for ten minutes at 37 °C. Stained cells were analyzed using the ACCURI C6 PLUS (BD Biosciences, San Jose, CA, USA) cytofluorimeter. Following doublet discrimination, gated events were then visualized on a log scale in FL2 585/40. Approximately 3000 events were acquired for each sample.

### 2.10. Reactive Oxygen Species (ROS) Detection

A total of 40,000 cells were seeded in 24-well plates. After 24 h, the growth medium was replaced with fresh medium containing the desired treatment, and the cells were left to grow for an additional 24 or 48 h. To assay the general oxidative activity, cells were stained with a 20 µM diacetyldichlorofluorescein (DCFH-DA) probe (Sigma-Aldrich) during the last two hours of treatment. Then, cells were detached by trypsin treatment and suspended in 300 µL of PBS for cytometry analysis. A 2 h treatment with t-BHP was used as a positive control. Following doublet discrimination, gated events were then visualized on a log scale in the FL1 530 BP channel to record the mean fluorescence intensity. Approximately 3000 events were acquired for each sample.

### 2.11. Phospho-Histone 3 Immunostaining 

A total of 400,000 cells were seeded in 6-well plates. After 24 h, the growth medium was replaced with fresh medium containing the desired treatment, and the cells were left to grow for an additional 24 or 48 h. Detection of mitotic cells was obtained by immunostaining cells with anti-phospho-histone H3 (Ser10) antibody (Thermo Fisher Scientific) according to the following protocol: Cells were detached by trypsin treatment, fixed as described for cell cycle analysis, and suspended in 0.5 mL PBS 1 × supplemented with 1% bovine serum albumin (BSA) and 0.2% Triton-100 for 15 min on ice. After centrifugation, cells were suspended in a 1:500 dilution of anti-phospho-histone H3 (Ser10) antibody in PBS 1 × plus 1% BSA for 2 h at room temperature. Cells were then washed in PBS 1 × plus 1% BSA and incubated with a 1:200 dilution of anti-rabbit Alexa Fluor 488 secondary antibody (Thermo Fisher Scientific) for 30 min at room temperature. After washing, cells were analyzed using an ACCURI C6 PLUS (BD Biosciences) flow cytometer. Doublet discrimination was manually carried out by plotting events on a linear scale according to the FSC signal area versus the FSC signal height. Gated events were then plotted on a log scale for the FL1 530 BP channel versus the FSC channel. Approximately 5000 events were acquired for each sample.

### 2.12. Transmission Electron Microscopy

A total of 400,000 cells were seeded into 6-well plates. After 24 h, the growth medium was replaced with fresh medium containing the desired treatment, and the cells were allowed to grow for an additional 24 or 48 h. Cells were then detached using trypsin, collected by centrifugation, and washed in PBS. The cell pellets were fixed in 2.5% glutaraldehyde (Electron Microscopy Sciences, Hatfield, PA, USA) in Na-cacodylate buffer for 1 h at 4 °C, post-fixed in 1% osmium tetroxide, and then dehydrated and embedded in epon-araldite resin. Ultrathin sections were prepared using a Reichert–Jung Ultracut ultramicrotome, placed on formvar carbon grids, and stained with 4% (*w*/*v*) uranyl acetate followed by 0.2% (*w*/*v*) lead citrate. The samples were then analyzed using a Jeol 100 SX electron microscope (Jeol, Milano, Italy).

### 2.13. Statistical Analysis

Student’s *t*-test or one-way ANOVA were applied to evaluate the statistical significance of differences between experimental classes. Differences were deemed statistically significant if the *p*-values were less than 0.05. Statistical significance was evaluated within each experiment. The consistency of results was verified in three independent experiments. In case different experimental treated classes did not share the same control, values for each class were all normalized versus the mean value of the corresponding control before being compared with each other. Everywhere not specified, the graphs and statistical analysis were carried out by means of Microsoft excel. EC50 values were calculated by using the Graphpad Prism program using nonlinear regression-[inhibitor] vs. response (3 parameters) analysis tool.

## 3. Results

### 3.1. D. japonica Mucus Exhibited Inhibitory Effect on the Growth of Human Carcinoma Cells

**Figure 2:**
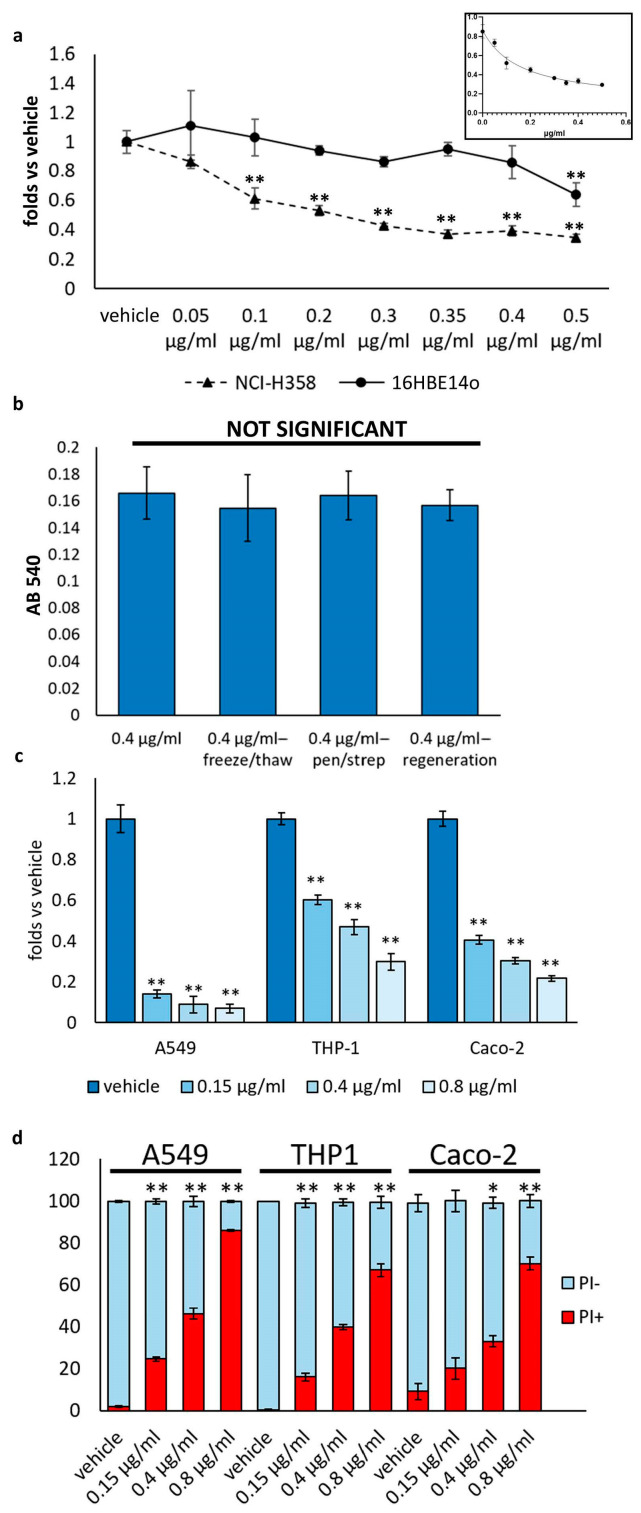
Effect of *D. japonica* Mucus Treatment on Cell Growth. (**a**) A representative experiment demonstrating the dose-dependent impact of mucus treatment on NCI-H358 and 16HBE14o cell numbers, assessed via crystal violet assay 48 h post-treatment. Each point reflects the mean value (±SD) of three independent samples, normalized to their respective controls. ** = *p* < 0.01, calculated using Student’s *t*-test. The inset displays EC50 values determined using the Graphpad Prism program. (**b**) This representative experiment illustrates the influence of various mucus preparations on NCI-H358 cell growth, evaluated through crystal violet assay 48 h post-treatment. Each bar represents the mean absorbance (±SD) of three independent samples. NS indicates not significant, determined by one-way ANOVA. (**c**) A representative experiment illustrating the dose-dependent impact of mucus treatment on cell numbers in A549, Caco-2, and THP-1 cells. Cell numbers were assessed using the crystal violet assay for A549 and Caco-2 cells, and direct counting for THP-1 cells, 48 h post-treatment. Each bar represents the mean value (±SD) of three independent samples, normalized to their respective controls. ** = *p* < 0.01, calculated by Student’s *t*-test. (**d**) Representative experiment, in which we observe the dose-dependent effect of mucus treatment on cell death, evaluated using the PI incorporation assay in A549, Caco-2, and THP-1 cells, 48 h post-treatment. Each bar represents the percentage of dead and alive cells (±SD) assessed in three independent samples. * = *p* < 0.05; ** = *p* < 0.01, determined by Student’s *t*-test.

To investigate the effect of *D. japonica* mucus preparation on cancer cells, we treated both normal 16HBE14o bronchial epithelial cells and NCI-H358 bronchioalveolar carcinoma cells with different concentrations of mucus ranging from 0.05 μg/mL to 0.5 μg/mL and evaluated cell growth after 24 and 48 h using the crystal violet assay, a technique that allows to monitor cell number and analyze growth curves. As depicted in Figure 2a, after 48 h of treatment, *D. japonica* mucus was more effective in reducing NCI-H358 growth compared to normal bronchial epithelial cells (16HBE14o), significantly affecting NCI-H358 cells in a concentration-dependent manner, starting from the concentration of 0.05 μg/mL, with an estimated EC50 value of 0.102 ± 0.08 μg/mL. The EC50 value for 16HBE14o was higher to 0.4 μg/mL. No significant difference with respect to control was observed 24 h after treatment by the crystal violet assay. These findings suggest that a treatment of 48 h with planarian mucus is able to inhibit cell growth of cancer cells more efficiently than normal counterparts.

Comparison between fresh and frozen mucus preparation (thawed a couple of times) revealed no significant difference in their ability to reduce NCI-H358 growth 48 h after treatment (Figure 2b), thus demonstrating that planarian mucus preparation can be stored for future use without compromising its anti-growth activity. Furthermore, the potential bacterial origin of the mucus component responsible for cytotoxic/cytostatic activity was ruled out by comparing the effect of mucus preparation obtained from planarians treated with 100 units/mL of penicillin and 100 μg/mL of streptomycin for one week with that of planarian mucus obtained from untreated planarians (Figure 2b). Finally, mucus preparation obtained from intact or 2 days regenerating planarians showed similar effects on NCI-H358 growth (Figure 2b), thus suggesting that the production of mucus components involved in the anti-growth activity is not modulated during the physiological regenerative process of planarians.

To assess whether *D. japonica* mucus preparation also affected different types of tumor cells, we analyzed the cell growth of A549 human non-small-cell lung cancer cells, Caco-2 human colorectal adenocarcinoma epithelial cells, and the THP-1 human leukemia monocytic cell line using crystal violet assay or cell counting. As shown in Figure 2c, mucus preparation significantly inhibited tumor cell growth in a dose-dependent manner.

### 3.2. Mucus Treatment Induces Both Cytostatic and Cytotoxic Effects on NCI-H358 Cells

**Figure 3:**
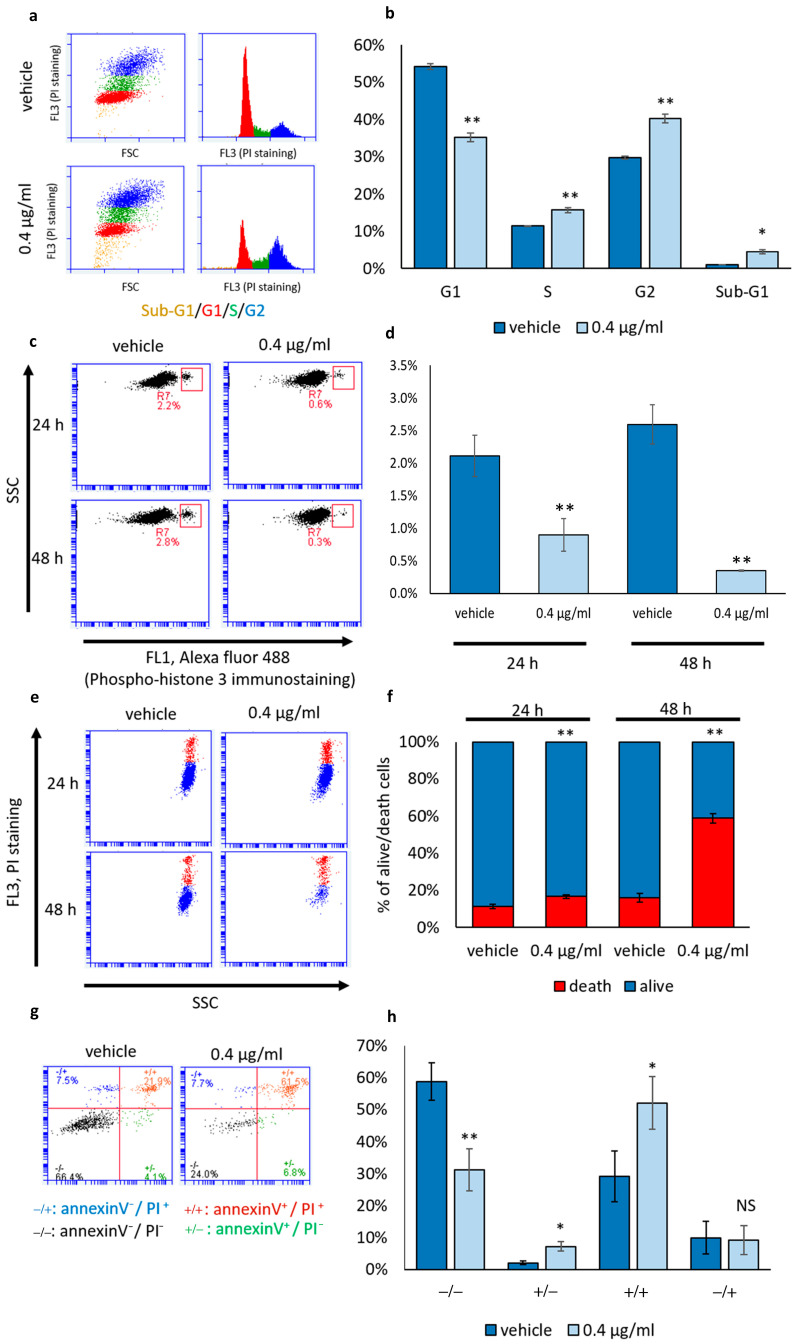
Effect of *D. japonica* Mucus Treatment on NCI-H358 Cell Cycle, Mitotic Index and Cell Death. (**a**) Representative cytometry plots showing the distribution of NCI-H358 cells across cell cycle phases, assessed 48 h post-treatment. (**b**) Graph illustrating the percentage of cells in different cell cycle phases, as evaluated in a representative experiment. Each bar represents the mean values (±SD) derived from three independent samples. * = *p <* 0.05; ** = *p <* 0.01, determined by Student’s *t*-test. (**c**) Representative cytometry plots showing the H3p positive mitotic cells (red box). (**d**) Graph depicting the percentage of H3p-positive cells, as assessed in a representative experiment. Each bar represents the mean values (±SD) derived from three independent samples. ** = *p <* 0.01, calculated by Student’s *t*-test. (**e**) Representative cytometry plots depicting the impact of mucus treatment on cell death, evaluated using the PI incorporation assay at 24 and 48 h post-treatment. Live cells are stained in blue; dead cells in red. (**f**) Graph illustrating the percentage of live and dead cells, as evaluated in a representative experiment by using the PI incorporation assay at 24 and 48 h post-treatment. Each bar represents the percentage of dead and alive cells (±SD) analyzed across three independent samples. ** = *p* < 0.01 determined by Student’s *t-*test. (**g**) Representative cytometry plots depicting the effect of 48 h mucus treatment on the percentage of annexinV+ cells, annexinV−/PI+ cells, PI+ cells and annexinV−/PI− cells in the annexin/PI incorporation assay. (**h**) Graph depicting the percentage of cells negative for both PI and annexinV (−/−); positive for PI and negative for annexinV (+/−); negative for PI and positive for annexinV (−/+); positive for PI and annexinV (+/+). Each bar represents the mean values (±SD) derived from three independent samples. * = *p <* 0.05; ** = *p <* 0.01, NS = not significant, determined by Student’s *t*-test.

To assess the impact of mucus preparation on NCI-H358 cell proliferation, we analyzed the distribution of cells in different phases of the cell cycle through DNA staining and flow cytometry (Figure 3). NCI-H358 cells were treated with mucus at the concentration of 0.4 μg/mL for 48 h, with cells treated with the vehicle alone serving as controls. As depicted in Figure 3a,b, a 48 h treatment with mucus altered the cell distribution across different phases of the cell cycle. Specifically, we observed an increase in the proportion of cells in the S phase and a significant arrest in the G2 phase, accompanied by a reduction in the percentage of cells in the G1 phase. Furthermore, analysis of the mitotic index by counting the percentage of cells positive for the mitotic marker phospho-histone H3 (Ser10), revealed a notable decrease in the percentage of mitotic cells in samples treated with mucus for 24 or 48 h (Figure 3c,d). These data support a cytostatic activity of mucus preparation.

Cell cycle analysis also unveiled a significant increase in the cell population with sub-G1 DNA content (Figure 3b), indicative of cell death. Accordingly, the propidium iodide (PI) incorporation assay—in which only dead cells with broken membranes are stained—showed a slight but significant increase in the percentage of dead cells 24 h after treatment and a substantial increase in the number of dead cells 48 h after treatment with mucus (Figure 3e,f). Additionally, as illustrated in Figure 2d, in all other tested cell lines, a higher percentage of dead cells was observed following 48 h of treatment with mucus compared to vehicle-treated samples.

Furthermore, the annexin V/PI assay, conducted 48 h after treatment was used to analyze the percentage of apoptotic and necrotic cells as well as the presence of secondary necrotic cells or cells undergoing alternative programmed cell death processes. Annexin V/PI staining revealed a slight but significant increase in the percentage of early apoptotic cells (annexin V+/PI−) in mucus-treated samples. However, in these samples, the majority of dead cells were positive for both annexin V and PI (annexin V+/PI+), secondary necrotic cells or cells undergoing alternative programmed necrotic cell death. Pure necrotic cells (annexin V−/PI+) were equally distributed between mucus-treated cells and corresponding controls (Figure 3g,h).

### 3.3. Mucus Treatment Induces Mixed Cell Death Modalities, Accumulation of Lipid Droplets and Mitochondrial Membrane Reorganization

**Figure 4:**
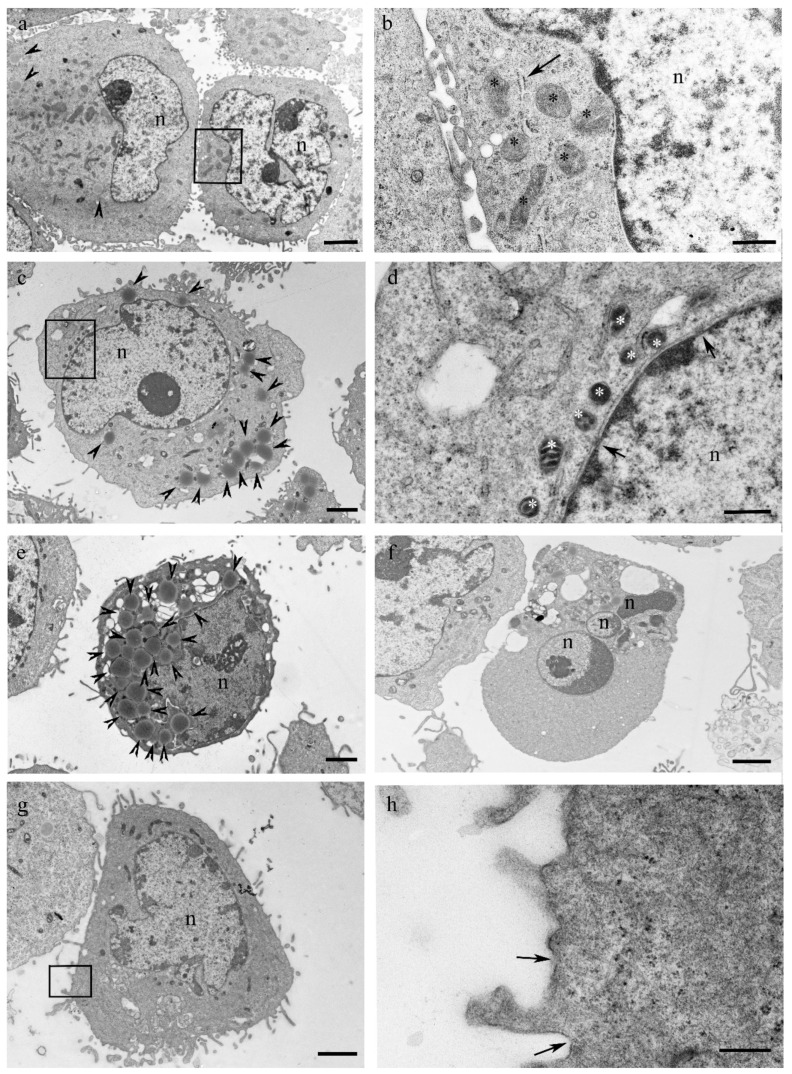
Effect of *D. japonica* Mucus Treatment on NCI-H358 Cell Morphology by Transmission Electron Microscopy. (**a**) Representative control NCI-H358 cells showing rare lipid droplets (arrowheads). (**b**) Magnification of the boxed area in A, showing a well-preserved cytoplasm containing some mitochondria (asterisks) with electrondense matrix and well-organized cristae, and a portion of the rough endoplasmatic reticulum (arrow). (**c**) Representative mucus-treated NCI-H358 cell showing several lipid droplets (arrowheads) in the cytoplasm. (**d**) Magnification of the boxed region in C showing some shrunken mitochondria (asterisks). The nuclear envelope is well-preserved as evidenced by the presence of nuclear pores (arrows). (**e**) Micrograph showing a mucus-treated NCI-H358 cell with a very high number of lipid droplets (arrowheads). (**f**) Representative mucus-treated NCI-H358 cell with a fragmented heterochromatic nucleus. (**g**) Representative mucus-treated NCI-H358 cell with damaged plasma membrane. (**h**) High magnification of the boxed area in h showing the presence of interruptions in the plasma membrane (arrows). n: nucleus. Scale bar is: 2 μm in (**a**,**c**,**e**,**f**,**g**); 500 nm in (**b**,**d**); 400 nm in (**h**).

As the presence of a high number of annexin V+/PI+ cells is a common feature of secondary necrosis, as well as programmed necrotic cell death as necroptosis [30], pyroptosis [31] and ferroptosis [32], we decided to deeply investigate the ultrastructural features of mucus-treated cells by electron microscopy (Figure 4).

According to annexin V/PI assay data, rare apoptotic cells with chromatin condensation and nuclear fragmentation were observable in mucus-treated samples (Figure 4f). However, most of the mucus-treated cells showed a nucleus with no chromatin condensation and a well-preserved nuclear envelope (Figure 4c,d). These cells, also showed a not-swelling cytoplasm containing shrunken mitochondria with a condensed mitochondrial membrane and reduced cristae, a high number of lipid droplets (Figure 4a–e) and the presence of plasma membrane interruptions (Figure 4g,h).

**Figure 5:**
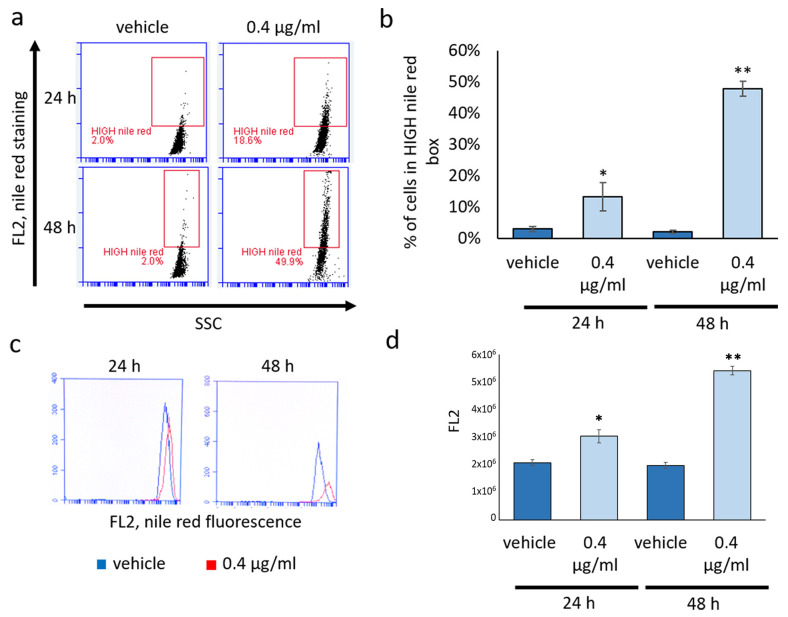
Effect of *D. japonica* Mucus Treatment on Lipid Droplets Evaluated by Nile Red Staining. (**a**) Representative cytometry plots displaying Nile red-stained NCI-H358 cells at 24 and 48 h post-treatment. (**b**) Graph illustrating the percentage of cells exhibiting high Nile red content (within the HIGH Nile red box) in a representative experiment. Each bar represents the mean values (±SD) evaluated in three independent samples. * = *p <* 0.05; ** = *p <* 0.01 calculated by Student’s *t*-test. (**c**) A representative cytometry graph showcases the distribution of Nile red fluorescence (FL2) in a vehicle-treated sample (blue line) and a mucus-treated sample (red line) at 24 and 48 h post-treatment. (**d**) The graph depicts the mean Nile red fluorescence (FL2) assessed in a representative experiment. Each bar represents the mean value (±SD) from three independent samples. * = *p <* 0.05; ** = *p <* 0.01 calculated by Student’s *t*-test.

A marked increase in lipid droplets following mucus treatment was further confirmed by flow cytometry quantification of Nile red staining at both 24 and 48 h (Figure 5).

**Figure 6:**
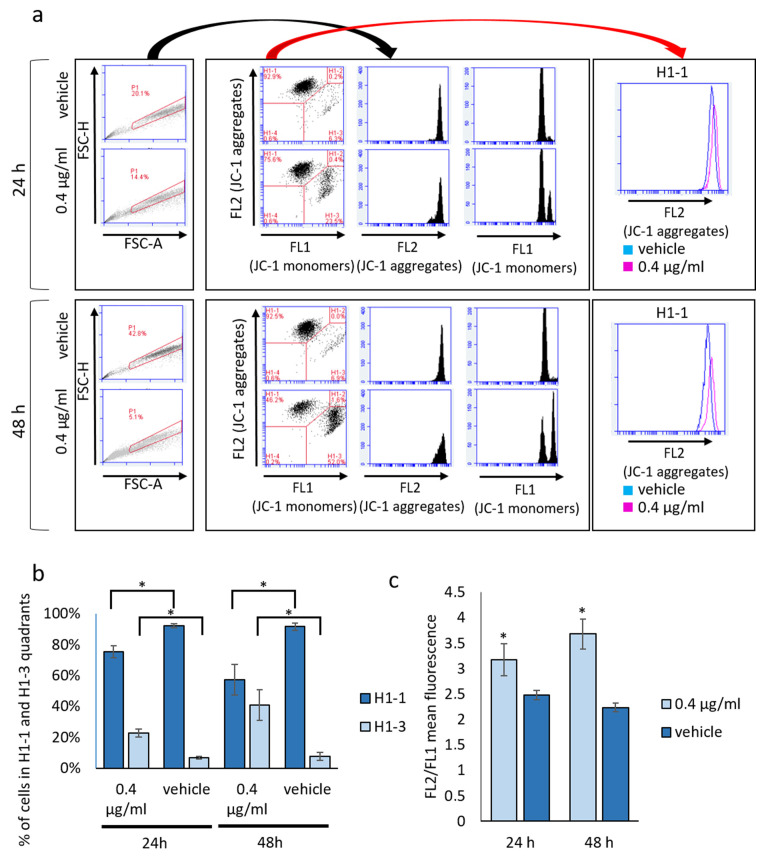
Effect of *D. japonica* Mucus Treatment on Mitochondrial Transmembrane Potential (ΔΨ). (**a**) Illustrative cytometry plots representing the impact of mucus treatment on ΔΨ, assessed using the JC1 staining assay at 24 and 48 h post-treatment. (**b**) Graph illustrating the percentage of cells in the H1-1 quadrant (polarized) and H1-3 quadrant (depolarized) quantified in a representative experiment. Each bar represents the mean value (±SD) evaluated across three independent samples. * = *p <* 0.05 calculated by Student’s *t*-test. (**c**) Graph depicting the FL2/FL1 ratio calculated for H1-1 cells in a representative experiment. Each bar represents the mean value (±SD) evaluated in three independent samples * = *p <* 0.05 calculated by Student’s *t*-test.

Moreover, in accordance with the major reorganization of the mitochondrial inner membrane observed by electron microscopy, the analysis of mitochondrial transmembrane potential (ΔΨ) by JC-1 staining revealed a bimodal modulation of ΔΨ in mucus-treated samples. Particularly, at both 24 and 48 h, a consistent increase in the percentage of cells with depolarized mitochondria (H1-3 quadrant of Figure 6a) is observable following mucus treatment (Figure 6b). Concomitantly, in mucus-treated samples, other cells (H1-1 quadrant of Figure 6a) show a significant increase in ΔΨ (hyperpolarized mitochondria). Consistently, the red/green (FL2/FL1) ratio of these cells is significantly higher in treated samples compared to vehicle samples (Figure 6c). Mitochondrial permeability transition pore (mPTP) opening does not appear to be involved in ΔΨ perturbation produced by mucus treatment, as co-treatment with bongkrekic acid (a well-known mPTP blocker) does not rescue mitochondrial depolarization and cell viability.

### 3.4. Cytotoxic and Not Cytostatic Effect Produced by Mucus Is Mediated by Reactive Oxygen Species

**Figure 7:**
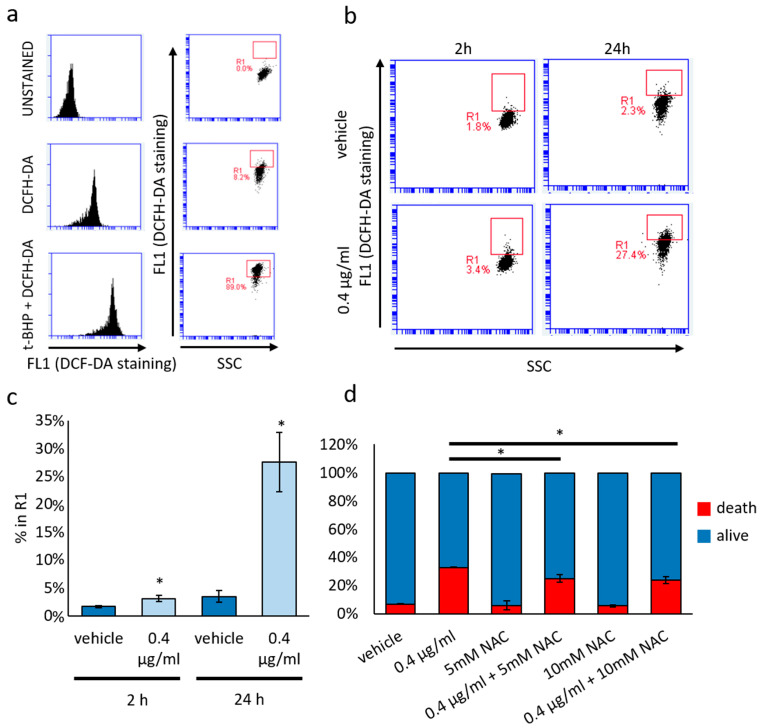
Effect of *D. japonica* Mucus Treatment on ROS Production. (**a**) Representative cytometry plots showing the experimental set-up in DCFH-DA staining experiments. T-BHP is a well-known pro-oxidant substrate. (**b**) Representative cytometry plots showing the effect of mucus treatment on DCFH-DA staining 2 and 24 h after treatment. (**c**) Graph depicting the percentage of cells in the R1 box quantified in a representative experiment. Each bar represents the mean value (±SD) evaluated in three independent samples. * *p <* 0.05 calculated by Student’s *t-*test. (**d**) Graph depicting the effect of the co-treatment with NAC on cell viability evaluated by PI incorporation assay in a representative experiment. Each bar represents the percentage of dead and alive cells (±SD) evaluated in three independent samples. * = *p <* 0.05 calculated by Student’s *t*-test.

To assess the involvement of ROS in mediating the cytotoxic activity of mucus, we quantified the ROS-mediated conversion of DCFH-DA into the highly fluorescent 2′,7′-dichlorofluorescein (DCF) (Figure 7). As depicted in Figure 7a–c, DCF green emission significantly increases, starting from 2 h of treatment, with a consistent increase observed in 24 h mucus-treated samples compared to controls. These data suggest that ROS increase precedes the cytotoxic effects, indicating their pivotal role in mucus-induced cell death.

**Figure 8:**
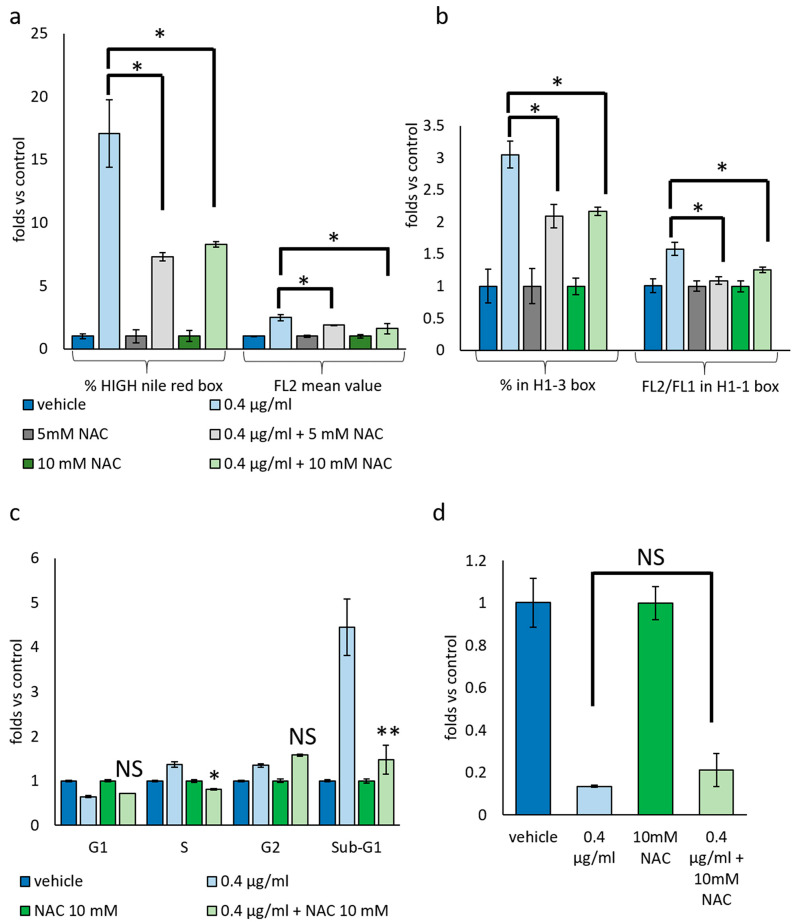
Effect of Antioxidant on *D. japonica* Mucus Treatment. (**a**) Graph depicting the effect of NAC co-treatment on lipid droplets accumulation evaluated by Nile red staining. (HIGH Nile red box, as for Figure 7). Each bar represents the mean value (±SD) evaluated in three independent samples normalized versus the correspondent control to which an arbitrary value of 1 has been attributed. * *p <* 0.05 calculated by Student’s *t*-test. (**b**) Graph depicting the effect of NAC co-treatment on ΔΨ modulation evaluated by JC1 staining (H1-1 and H1-3 boxes as for Figure 8). Each bar represents the mean value (±SD) evaluated in three independent samples normalized versus the correspondent control to which an arbitrary value of 1 has been attributed. * = *p <* 0.05 calculated by Student’s *t*-test. (**c**) Graph depicting the effect of NAC co-treatment on cell cycle 48 h after treatment. Each bar represents the mean value (±SD) evaluated in three independent samples normalized versus the correspondent control to which an arbitrary value of 1 has been attributed. * = *p <* 0.05; ** = *p <* 0.01; NS = not significant calculated by Student’s *t*-test. (**d**) Graph depicting the effect of NAC co-treatment on mitotic index 48 h after treatment. Each bar represents the mean value (±SD) evaluated in three independent samples normalized versus the correspondent control to which an arbitrary value of 1 has been attributed. NS = not significant, calculated by Student’s *t*-test.

To confirm this hypothesis, we co-treated cells with mucus and the potent antioxidant NAC at both 5 and 10 mM concentrations. As illustrated in Figure 7d, mucus effects on cell viability are mitigated by NAC co-treatment in a dose-dependent manner. Moreover, NAC partially rescues the accumulation of lipid droplets (Figure 8a) and the bimodal modulation of ΔΨ (Figure 8b) induced by 0.4 μg/mL mucus treatment. Lipid content was also reduced by using 500 μM ascorbic acid as antioxidant.

However, not all the effects produced by mucus treatment on cell proliferation are affected by NAC co-treatment. Specifically, NAC completely rescues the mucus-induced S-phase slowdown, mitigates the accumulation of sub-G1 cells, and does not affect G2 arrest and mitotic index drop (Figure 8c,d).

## 4. Discussion

Natural bioactive molecules provide an almost inexhaustible reservoir for discovering new compounds with potential anti-tumor activity. In this context, we succeeded in developing a protocol for collecting the mucus fraction from *D. japonica* live specimens, which can then be reintegrated into the colony without adverse effects. After the procedure, we obtained a mucus preparation with any residual traces of NAC not exceeding 0.25%. Bacterial contaminants, if present, were unable to grow in the presence of penicillin/streptomycin at the concentrations used for cell culture, ensuring that the mucus preparation could be used without the risk of contamination.

The mucus inhibits the growth of several tumor cell lines. Among them, we focused our attention on the NCI-H358 human bronchioalveolar carcinoma cell line, for which a direct comparison with a corresponding non-tumoral cell line (16HBE14o human bronchial epithelial cell) suggested a preferential anti-tumor effect. In planarian ecology, the ability of mucus to inhibit cell growth might confer a significant advantage in defense against predators or parasites. An innate immune protection role has been already proposed for anticancer peptides identified in the mucus of the *Achatina fulica* snail [25]. Moreover, it has been demonstrated that *Actinia equine* mucus possesses cytotoxic activity against the K562 cancer cell line and antibacterial activity [26]. However, the potential of *D. japonica* mucus to inhibit bacterial or fungal growth has yet to be tested and is beyond the scope of this paper.

The reduction in tumor cell viability produced by mucus treatment includes both cytotoxic and cytostatic effects. We observed alterations in the cell cycle as well as a sustained increase in cell death. The cell cycle of mucus-treated cells was characterized by an increase in the proportion of cells in the S and G2 phases, along with a decrease in the mitotic index. Cytotoxic effects encompass the activation of different cell death programs. Some mucus-treated cells activate apoptosis, while most of the cells exhibited morphological changes that could be ascribable to a ferroptosis-like cell death modality, i.e., shrunken mitochondria and membrane damage [32,33]. Moreover, in mucus-treated cells, a significant accumulation of lipid droplets was found. This is consistent with the uncontrolled lipid peroxidation known to drive ferroptotic cell death [34]. Indeed, a double-edged sword in regulating ferroptosis has been proposed for lipid droplets as they can act as stress response organelles and suppress ferroptosis [35,36] or act as a source of lipid oxidation and promote it [37].

Mitochondrial ultrastructure alteration in mucus-treated samples is accompanied by an atypical modulation of the membrane potential (ΔΨ). Observing the data, it is clear that highly depolarized cells accumulate in a time-dependent manner, correlating with increased cell death. However, we also observed that in mucus-treated samples, cells with unaffected ΔΨ exhibited higher fluorescence levels of JC1 aggregates, suggesting mitochondrial membrane hyperpolarization. This bimodal modulation of ΔΨ requires further investigation to be fully explained; however, one possible hypothesis is that mucus treatment induces a rapid increase in ΔΨ, followed by a subsequent collapse. Early mitochondrial hyperpolarization preceding ΔΨ collapse has been previously documented during programmed cell death [38,39,40,41]. However, although different possible explanations were suggested, the precise mechanism(s) involved in mitochondrial hyperpolarization remains unclear.

Most of the cell responses induced by mucus are triggered by an increase in the amount of ROS, which is an early event in mucus-treated cells. The common antioxidant NAC, at concentrations of 5–10 mM, was able to counteract cellular alterations induced by mucus, including lipid droplet accumulation, modulation of mitochondrial membrane potential, and the slowdown of the S-phase of the cell cycle, as well as reducing the number of dead cells. However, G2 arrest and the drop in mitotic index were independent of ROS, suggesting multiple scenarios as follows: (i) different toxicants affecting multiple cellular pathways are produced in mucus secretion; (ii) a single toxicant exhibits pleiotropic activity by mediating both ROS production and cell cycle arrest; (iii) cell cycle arrest indirectly induces ROS accumulation [42], which is responsible for additional cytotoxic effects. Although none of these hypotheses can be ruled out, the pleiotropic activity of a single toxicant is consistent with several literature data reports on natural products with anticancer activity [43,44].

Due to the molecular weight cut-off used in the preparative procedure to obtain the planarian mucus fraction, the active compound(s) responsible for its anticancer activity is (are) expected to have a molecular weight higher than 3000 daltons. This led to the exclusion of small molecule drugs and to suggest the presence of larger molecules, such as antimicrobial peptides that exhibit anticancer activity, the so-called anticancer peptides [45,46]. Anticancer peptides are small, cationic molecules composed of 5–50 amino acids, playing a crucial role in the immune system by defending against external pathogens like fungi, bacteria and viruses. Over 750 distinct anticancer peptides have been identified across a wide range of organisms, from insects and plants to animals, including humans [47]. Several bioactive molecules extracted in mucus from different organisms are anticancer peptides that can act specifically in tumor cells but not in healthy mammalian cells [25,48], inducing cell death probably through mechanisms of mitochondrial or plasma membrane invasion [49]. The ability of antioxidants to mitigate lipid droplet accumulation in mucus-treated cells is consistent with the physiological role of these organelles, which includes reducing cell damage induced by lipid radicals [37,50].

At the mitochondrial level, scavenging ROS reduces both hyperpolarization and depolarization events. Moreover, bongkrekic acid, the mitochondrial permeability transition pore blocker, did not rescue ΔΨ modulation or cell death, suggesting that mitochondria are a target of ROS produced in an extramitochondrial cellular district, such as the plasma membrane or endoplasmic reticulum.

At this time, we do not know the exact composition of planaria mucus or which component might be responsible for the effects it produces. Thus, we can only cautiously hypothesize that a pleiotropic mucus peptide(s) directly or indirectly triggers, on one side, cell cycle arrest, and on the other, a pro-radical activity which induces lipid peroxidation. Lipid peroxides might lead to mitochondrial damage, and lipid droplet accumulation might be a scavenging attempt from lipid radicals. Cell cycle arrest itself might be involved in stimulating lipid droplet accumulation as a failed attempt to escape ferroptosis [51].

Most natural products of animal origin are produced by symbiotic microorganisms, especially bacteria [52,53]. In our case, pretreatment of planarians with penicillin and streptomycin did not diminish the anticancer effect of mucus, suggesting that planarians directly produce the active chemical substance(s). However, we cannot rule out the possibility of active substance production by penicillin/streptomycin-resistant bacteria or of fungal origin.

To the best of our knowledge, this is the first evidence that planarian mucus exhibits cytotoxic and cytostatic effects. *D. japonica* mucus inhibits the growth of human bronchioalveolar carcinoma cells by activating multiple cytotoxic and cytostatic mechanisms, suggesting a pleiotropic activity that could be advantageous in combating multifactorial diseases such as cancer. This work opens up numerous research perspectives, primarily aimed at identifying the composition of the mucus and its active components. This step is essential for discovering novel bioactive molecules that could inspire chemical synthesis or be purified to ensure quality control, ultimately enabling the development of strategies for the potential therapeutic use of mucus.

## Data Availability

All data supporting the findings of this study are available within the paper.

## References

[1] Newman D.J., Cragg G.M. (2016). Natural products as sources of new drugs from 1981 to 2014. J. Nat. Prod..

[2] Corey E.J., Barton D.H.R., Nakanishi K., Meth-Cohn O. (1999). Editorial review for comprehensive natural products chemistry. Comprehensive Natural Products Chemistry.

[3] Atanasov A.G., Zotchev S.B., Dirsch V.M., Supuran C.T., International Natural Product Sciences Taskforce (2021). Natural products in drug discovery: Advances and opportunities. Nat. Rev. Drug Discov..

[4] Feher M., Schmidt J.M. (2003). Property distributions: Differences between drugs, natural products, and molecules from combinatorial chemistry. J. Chem. Inf. Comput. Sci..

[5] Yuan H., Ma Q., Ye L., Piao G. (2016). The Traditional Medicine and Modern Medicine from Natural Products. Molecules.

[6] Cechinel F.V. (2018). Natural Products as Source of Molecules with Therapeutic Potential: Research & Development, Challenges and Perspectives.

[7] Cragg G.M., Pezzuto J.M. (2016). Natural Products as a Vital Source for the Discovery of Cancer Chemotherapeutic and Chemopreventive Agents. Med. Princ. Pract..

[8] Chaachouay N., Zidane L. (2024). Plant-Derived Natural Products: A Source for Drug Discovery and Development. Drugs Drug Candidates.

[9] Nasim N., Sandeep I.S., Mohanty S. (2022). Plant-derived natural products for drug discovery: Current approaches and prospects. Nucleus.

[10] Carroll A.R., Copp B.R., Davis R.A., Keyzers R.A., Prinsep M.R. (2023). Marine natural products. Nat. Prod. Rep..

[11] Rossi L., Salvetti A. (2019). Planarian stem cell niche, the challenge for understanding tissue regeneration. Semin. Cell Dev. Biol..

[12] Alessandra S., Rossi L. (2019). Planarian Stem Cell Heterogeneity. Adv. Exp. Med. Biol..

[13] Ireland D., Bochenek V., Chaiken D., Rabeler C., Onoe S., Soni A., Collins E.S. (2020). Dugesia japonica is the best suited of three planarian species for high-throughput toxicology screening. Chemosphere.

[14] Hagstrom D., Cochet-Escartin O., Zhang S., Khuu C., Collins E.M. (2015). Freshwater Planarians as an Alternative Animal Model for Neurotoxicology. Toxicol. Sci..

[15] Hagstrom D., Cochet-Escartin O., Collins E.M. (2016). Planarian brain regeneration as a model system for developmental neurotoxicology. Regeneration.

[16] Suleiman S., Di Fiore R., Cassar A., Formosa M.M., Calleja-Agius J., Schembri-Wismayer P. (2020). Anticancer effects of an extract from a local planarian species on human acute myeloid leukemia HL-60 cells in vitro. Biomed. Pharmacother..

[17] Pedersen K.J. (1963). Slime-secreting cells of planarians. Ann. N. Y. Acad. Sci..

[18] Hayes M.J. (2017). Sulphated glycosaminoglycans support an assortment of planarian rhabdite structures. Biol. Open.

[19] Gao L., Li A., Li N., Liu X., Deng H., Zhao B., Pang Q. (2017). Innate and intrinsic immunity in planarians. Invertebr. Surviv. J..

[20] Martin G.G. (1978). A New Function of Rhabdites: Mucus Production for Ciliary Gliding. Zoomorphologie.

[21] Wilden B., Majdi N., Kuhlicke U., Neu T.R., Traunspurger W. (2019). Flatworm mucus as the base of a food web. BMC Ecol..

[22] Pedersen K.J. (1953). Some features of the fine structure and histochemistry of planarian subepidermal gland cells. Z. Für Zellforsch. Und Mikrosk. Anat..

[23] Bowen I.D., Ryder T.A., Winters C. (1975). The distribution of oxidizable mucosubstances and polysaccharides in the planarian Polycelis tenuis Iijima. Cell Tissue Res..

[24] Bocchinfuso D.G., Taylor P., Ross E., Ignatchenko A., Ignatchenko V., Kislinger T., Pearson B.J., Moran M.F. (2012). Proteomic profiling of the planarian Schmidtea mediterranea and its mucous reveals similarities with human secretions and those predicted for parasitic flatworms. Mol. Cell. Proteom..

[25] E-Kobon T., Thongararm P., Roytrakul S., Meesuk L., Chumnanpuen P. (2015). Prediction of anticancer peptides against MCF-7 breast cancer cells from the peptidomes of Achatina fulica mucus fractions. Comput. Struct. Biotechnol. J..

[26] Stabili L., Schirosi R., Parisi M.G., Piraino S., Cammarata M. (2015). The Mucus of Actinia equina (Anthozoa, Cnidaria): An Unexplored Resource for Potential Applicative Purposes. Mar. Drugs.

[27] Balestrini L., Di Donfrancesco A., Rossi L., Marracci S., Isolani M.E., Bianucci A.M., Batistoni R. (2017). The natural compound sanguinarine perturbs the regenerative capabilities of planarians. Int. J. Dev. Biol..

[28] Masuko T., Minami A., Iwasaki N., Majima T., Nishimura S., Lee Y.C. (2005). Carbohydrate analysis by a phenol-sulfuric acid method in microplate format. Anal. Biochem..

[29] Smiley S.T., Reers M., Mottola-Hartshorn C., Lin M., Chen A., Smith T.W., Steele G.D., Chen L.B. (1991). Intracellular heterogeneity in mitochondrial membrane potentials revealed by a J-aggregate-forming lipophilic cation JC-1. Proc. Natl. Acad. Sci. USA.

[30] Pietkiewicz S., Schmidt J.H., Lavrik I.N. (2015). Quantification of apoptosis and necroptosis at the single cell level by a combination of Imaging Flow Cytometry with classical Annexin V/propidium iodide staining. J. Immunol. Methods.

[31] Miao E.A., Rajan J.V., Aderem A. (2011). Caspase-1-induced pyroptotic cell death. Immunol. Rev..

[32] Demuynck R., Efimova I., Naessens F., Krysko D.V. (2021). Immunogenic ferroptosis and where to find it?. J. Immunother. Cancer.

[33] Miyake S., Murai S., Kakuta S., Uchiyama Y., Nakano H. (2020). Identification of the hallmarks of necroptosis and ferroptosis by transmission electron microscopy. Biochem. Biophys. Res. Commun..

[34] Dixon S.J., Lemberg K.M., Lamprecht M.R., Skouta R., Zaitsev E.M., Gleason C.E., Patel D.N., Bauer A.J., Cantley A.M., Yang W.S. (2012). Ferroptosis: An iron-dependent form of nonapoptotic cell death. Cell.

[35] Beatty A., Singh T., Tyurina Y.Y., Tyurin V.A., Samovich S., Nicolas E., Maslar K., Zhou Y., Cai K.Q., Tan Y. (2021). Ferroptotic cell death triggered by conjugated linolenic acids is mediated by ACSL1. Nat. Commun..

[36] Zou Y., Palte M.J., Deik A.A., Li H., Eaton J.K., Wang W., Tseng Y.Y., Deasy R., Kost-Alimova M., Dančík V. (2019). A GPX4-dependent cancer cell state underlies the clear-cell morphology and confers sensitivity to ferroptosis. Nat. Commun..

[37] Danielli M., Perne L., Jarc Jovičić E., Petan T. (2023). Lipid droplets and polyunsaturated fatty acid trafficking: Balancing life and death. Front. Cell Dev. Biol..

[38] Oropesa M., de la Mata M., Maraver J.G., Cordero M.D., Cotán D., Rodríguez-Hernández A., Domínguez-Moñino I., de Miguel M., Navas P., Sánchez-Alcázar J.A. (2011). Apoptotic microtubule network organization and maintenance depend on high cellular ATP levels and energized mitochondria. Apoptosis.

[39] Kim J.M., Bae H.R., Park B.S., Lee J.M., Ahn H.B., Rho J.H., Yoo K.W., Park W.C., Rho S.H., Yoon H.S. (2003). Early mitochondrial hyperpolarization and intracellular alkalinization in lactacystin-induced apoptosis of retinal pigment epithelial cells. J. Pharmacol. Exp. Ther..

[40] Li P.F., Dietz R., von Harsdorf R. (1999). p53 regulates mitochondrial membrane potential through reactive oxygen species and induces cytochrome c-independent apoptosis blocked by Bcl-2. EMBO J..

[41] Marzo I., Brenner C., Zamzami N., Jürgensmeier J.M., Susin S.A., Vieira H.L., Prévost M.C., Xie Z., Matsuyama S., Reed J.C. (1998). Bax and adenine nucleotide translocator cooperate in the mitochondrial control of apoptosis. Science.

[42] Rowe L.A., Degtyareva N., Doetsch P.W. (2008). DNA damage-induced reactive oxygen species (ROS) stress response in Saccharomyces cerevisiae. Free Radic. Biol. Med..

[43] Lagunin A., Filimonov D., Poroikov V. (2010). Multi-targeted natural products evaluation based on biological activity prediction with PASS. Curr. Pharm. Des..

[44] Muhammad N., Usmani D., Tarique M., Naz H., Ashraf M., Raliya R., Tabrez S., Zughaibi T.A., Alsaieedi A., Hakeem I.J. (2022). The Role of Natural Products and Their Multitargeted Approach to Treat Solid Cancer. Cells.

[45] Chinnadurai R.K., Khan N., Meghwanshi G.K., Ponne S., Althobiti M., Kumar R. (2023). Current research status of anti-cancer peptides: Mechanism of action, production, and clinical applications. Biomed. Pharmacother..

[46] Gaspar D., Veiga A.S., Castanho M.A. (2013). From antimicrobial to anticancer peptides. A review. Front. Microbiol..

[47] Reddy K.V., Yedery R.D., Aranha C. (2004). Antimicrobial peptides: Premises and promises. Int. J. Antimicrob. Agents.

[48] Matusiewicz M., Kosieradzka I., Niemiec T., Grodzik M., Antushevich H., Strojny B., Gołębiewska M. (2018). In Vitro Influence of Extracts from Snail Helix aspersa Müller on the Colon Cancer Cell Line Caco-2. Int. J. Mol. Sci..

[49] Harris F., Dennison S.R., Singh J., Phoenix D.A. (2013). On the selectivity and efficacy of defense peptides with respect to cancer cells. Med. Res. Rev..

[50] Shyu P., Wong X.F.A., Crasta K., Thibault G. (2018). Dropping in on lipid droplets: Insights into cellular stress and cancer. Biosci. Rep..

[51] Lee H., Horbath A., Kondiparthi L., Meena J.K., Lei G., Dasgupta S., Liu X., Zhuang L., Koppula P., Li M. (2024). Cell cycle arrest induces lipid droplet formation and confers ferroptosis resistance. Nat. Commun..

[52] Piel J. (2009). Metabolites from symbiotic bacteria. Nat. Prod. Rep..

[53] König G.M., Kehraus S., Seibert S.F., Abdel-Lateff A., Müller D. (2006). Natural products from marine organisms and their associated microbes. ChemBioChem.

